# National survey of pediatric services available in US emergency departments

**DOI:** 10.1186/1865-1380-6-13

**Published:** 2013-04-24

**Authors:** Ashley F Sullivan, Susan A Rudders, Amanda L Gonsalves, Anne P Steptoe, Janice A Espinola, Carlos A Camargo

**Affiliations:** 1 Department of Emergency Medicine, Massachusetts General Hospital, 326 Cambridge Street, Suite 410, Boston, MA 02114, USA; 2 Department of Pediatrics, Hasbro Children’s Hospital, 593 Eddy Street, Providence, RI 02903, USA

**Keywords:** Accident and emergency, Health services research

## Abstract

**Background:**

Children account for nearly 20% of all US emergency department (ED) visits, yet previous national surveys found that many EDs lack specialized pediatric care. In response, a 2001 joint policy statement recommended resources needed by EDs for effective pediatric emergency care delivery. We sought to update and enhance previous estimates of pediatric services available in US EDs.

**Methods:**

We administered a telephone survey to a 5% random sample (*n* = 279) of all US EDs from the 2007 National Emergency Department Inventory-USA. The survey collected data on local capabilities (including typical management of three clinical scenarios) and prevalence of a coordinator for pediatric emergency care. We used descriptive statistics to summarize data. Multivariable logistic regression was used to examine the association between survey respondent and ED characteristics as well as the presence of a coordinator for pediatric emergency medicine.

**Results:**

Data were collected from 238 hospitals (85% response rate). A minority of hospitals had pediatric departments (36%) or intensive care units (12%). The median annual number of ED visits by children was 3,870 (interquartile range 1,500–8,800). Ten percent of hospitals had a separate pediatric ED; only 17% had a designated pediatric emergency care coordinator. Significant positive predictors of a coordinator were an ED pediatric visit volume of ≥1 patient per hour and urban location. Most EDs treated only mild-to-moderate cases of childhood bronchiolitis and asthma exacerbation (77% and 65%, respectively). Less than half (48%) of the hospitals reported the ability to surgically manage a child with acute appendicitis.

**Conclusion:**

We found little change in pediatric emergency services compared to earlier estimates. Our study results suggest a continued need for improvements to ensure access to emergency care for children.

## Background

Pediatric health emergencies occur commonly, and most emergency departments (EDs) routinely care for patients of all ages. In 2007, an estimated 20% of the 117 million ED visits in the US were by children; most of these visits were to non-pediatric EDs [1]. Due to children’s unique health care needs, the outcome of these visits depends greatly on the availability of prompt access to medical care providers trained in pediatrics with proper equipment and facilities [2].

Unfortunately, previous national studies have found major shortcomings in US EDs’ preparedness to treat children [3-5], resulting in the potential for suboptimal care. Several national guidelines have attempted to address these deficiencies [6,7]. In 2001, on the heels of a statement from the Institute of Medicine (IOM) revealing inadequate integration of pediatric services into the emergency health care system [8], joint policy guidelines were issued on the care of children in the ED [6]. These guidelines (updated in 2009) are intended for all EDs that care for children, are open 24 h per day, 7 days per week, and have a physician continuously on duty. The guidelines included recommendations that EDs should: (1) appoint coordinators for the emergency care of children, (2) have age-appropriate equipment/medications/supplies, and (3) have protocols in place to facilitate all aspects of emergency care for children. Almost a decade after the dissemination of these guidelines, we sought to update the status of pediatric emergency care in the US.

## Methods

This national survey was performed in 2009 by the Emergency Medicine Network [9]. Trained research assistants administered a 10-min telephone survey to a 5% simple random sample (*n* = 279) of all 4,874 EDs in the 2007 National Emergency Department Inventory (NEDI)-USA database [10]. The NEDI-USA database includes a comprehensive listing of all hospital-based or hospital-affiliated EDs in the US. Methods for derivation of NEDI-USA have been previously described [11]. Briefly, Emergency Medicine Network staff compile NEDI-USA through original data collection and integration of information from a variety of sources (e.g., Verispan Hospital Market Profiling Solution, American Hospital Association Annual Survey Database, Flex Monitoring Team, and Association of American Medical Colleges). EDs were defined as emergency care facilities open 24 h per day, 7 days per week. We excluded EDs in US territories and outlying areas (e.g., Puerto Rico, Guam, US Virgin Islands), specialty hospitals (e.g., mental health, orthopedic, or rehabilitation hospitals), federal hospitals (e.g., Veterans Affairs and Indian Health Service hospitals), and college infirmaries. Such facilities and others (e.g., correctional centers) may lack formal EDs, or they are not necessarily available for use by the general public.

The content of the survey (see Additional file 1) was based on the 2001 and updated 2009 guidelines for emergency pediatric services developed by the American Academy of Pediatrics, the American College of Emergency Physicians, and the Emergency Nurses Association [6,7]. Specifically, the survey consisted of 16 questions to assess general characteristics of the medical facility, pediatric services available, staffing, and typical management practices for three common pediatric complaints (bronchiolitis, asthma exacerbation, and acute appendicitis). Although previous surveys did not examine these pediatric complaints, we chose bronchiolitis because it is a leading cause of acute hospitalization among US infants [12,13]; asthma because it is a common medical condition that frequently presents to the ED and for which there are national management guidelines [1,14]; and appendicitis because it is a common surgical condition [15].

We used Stata 10.1 (StataCorp, College Station, TX) to summarize data using descriptive statistics. Multivariable logistic regression was used to examine the association between survey respondent and ED characteristics, and the presence of a coordinator for pediatric emergency medicine. A coordinator was defined as a physician or nurse identified to coordinate pediatric emergency medicine in the ED. Logistic regression results are reported as odds ratios with 95% confidence intervals (CIs). All *P* values are two-tailed, with *P* < 0.05 considered statistically significant. The Partners Human Research Committee classified this study as exempt. Consent was implied through voluntary completion of the survey.

## Results

Data were collected from 238 hospitals (85% response rate). Respondents included ED nurses (38%), ED nurse managers (23%), ED directors (18%), and other members of the ED staff (21%), such as emergency physicians and administrators. Approximately one-third (36%; 95% CI 30-43%) of the hospitals surveyed had a pediatric inpatient department; a smaller percentage (12%; 95% CI 8-17%) had a dedicated pediatric intensive care unit. We examined the association between presence of inpatient pediatric inpatient services (yes/no) and volume of pediatric ED care and found that those with inpatient pediatric care saw a median of 9,500 ED visits while those without saw a median of 2,300 ED visits (*P* < 0.001). The median number of ED beds per department was 13 (interquartile range [IQR], 6–26) and the median number of visits per year per department by children less than 18 years old was 3,870 (IQR, 1,500−8,800). Participating EDs had a median annual visit volume of 20,000 (IQR, 9,000−42,000). This annual visit volume was similar to the national median of 18,841 (IQR, 7,804−35,170), as calculated based on 2007 NEDI-USA data. Similarly, most participating EDs were in urban areas (55%), which is similar to the location of EDs nationally (58% urban).

In EDs that saw both adults and children (i.e., excluding EDs that reported seeing children only), a minority (10%) had a separate pediatric ED; however, there was a pediatrician consultant available in most EDs (61%). In EDs with a pediatrician consultant, the consultant was available 24 h per day, 7 days per week, in 92% of facilities. Pediatrician consultants arrived, on average, in 0–29 min in 68% of EDs, in 30–59 min in 30% of EDs, and in 60 min or more in 2% of EDs. In hospitals with a pediatric inpatient department or a dedicated pediatric intensive care unit, pediatrician consultants arrived, on average, in 0–29 min in 73% of EDs, in 30–59 min in 23% of EDs, and in 60 min or more in 4% of EDs. In hospitals without a pediatric inpatient department or a dedicated pediatric intensive care unit, pediatrician consultants arrived, on average, in 0–29 min in 59% of EDs, in 30–59 min in 39% of EDs, and in 60 min or more in 2% of EDs. Only 17% (95% CI 12-22%) of EDs reported having a designated physician or nurse coordinator for pediatric emergency care. Among sites with a coordinator, 60% (95% CI 41-73%) reported having both a physician and nurse coordinator, 18% (95% CI 7-33%) reported a physician coordinator, and 25% (95% CI 13-41%) reported a nurse coordinator. In a multivariable logistic regression model, an ED pediatric visit volume of ≥1 patient per hour (i.e., ≥8,760 visits per year) was a significant positive predictor of the presence of a designated coordinator for pediatric emergency care (Table 1). EDs in rural areas (both adjacent and non-adjacent to an urban area) were significantly less likely than EDs in urban areas to have a designated coordinator for pediatric emergency care.

**Table 1:** Multivariable logistic regression predicting the presence of an identified coordinator for pediatric emergency medicine

**Characteristics**	**Odds ratio**	**95% Confidence interval**		***P***
**ED pediatric visit volume (patients/hour)**				
<1	1.00	Reference		–
1.0-1.9	2.64	1.00	7.01	0.05
2.0-2.9	9.33	2.25	38.65	0.002
≥3	23.37	6.44	84.85	<0.001
**Urban/rural status**				
Urban	1.00	Reference		–
Rural, adjacent to urban	0.14	0.03	0.66	0.01
Rural, not adjacent to urban	0.10	0.01	0.81	0.03

Abbreviations: *ED*, emergency department.

A minority of EDs reported having inpatient resources needed to care for children with common pediatric complaints of all severity levels (Table 2). Specifically, 21% (95% CI 16-26%) of EDs reported that their facility was able to care for a 6 month old with bronchiolitis of any severity level, and one-third (33%; 95% CI 27%-39%) of EDs reported being able to provide care for a 6 year old with asthma exacerbation of any severity level. Approximately half (48%; 95% CI 42%-55%) of the hospitals were able to surgically manage a child with acute appendicitis in their facility.

**Table 2:** Management of common pediatric complaints in the emergency department

	***n***	**%**
*6 month old with bronchiolitis*		
Yes, all severity levels; severe cases receive inpatient care at hospital	49	21
Yes, mild to moderate cases only (outpatient care); severe cases transferred to another hospital	182	76
No, does not manage on site	7	3
*6 year old with an asthma exacerbation*		
Yes, all severity levels; severe cases receive inpatient care at respondent hospital	78	33
Yes, mild to moderate cases only (outpatient care); severe cases transferred to another hospital	154	65
No, does not manage on site	6	2
*6 year old with acute appendicitis*		
Yes, evaluate and transfer to the OR at hospital	115	48
Yes, evaluate and transfer to the OR if surgeon was available/comfortable	5	2
Yes, evaluate, but transfer to another hospital for surgery	112	47
No, does not manage on site	6	3

Abbreviations: *OR*, operating room.

## Discussion

Children who require emergency care have unique needs, including practitioners trained in the care of children, specialized equipment, and appropriate resources for potential hospitalization and/or surgical intervention. Previous studies have described poor concordance with national guidelines that are aimed at improving our ability to provide emergency care for children [4,5]. When comparing data from our survey to earlier national studies [3,5], we demonstrate little improvement across several basic markers of the availability of pediatric services at hospitals (Table 3). Compliance with some of the recommended guidelines for care of children in the ED has not improved. Specifically, there has been no significant increase in facilities with dedicated pediatric EDs or inpatient pediatric services since a 2001 publication [3]. The availability of a pediatric consultant to the ED also has remained the same. In addition, despite numerous calls for the appointment of a designated coordinator for pediatric emergency care, in two joint consensus statements [6,7] and the 2006 report from the IOM [16], the proportion of EDs with a pediatric coordinator (13% physician coordinator; 14% with a nurse coordinator) is virtually unchanged from findings in a 2003 survey [5]. Finally, we found that most EDs report that their facilities are not able to treat all severity levels of a few common pediatric medical/surgical emergencies (bronchiolitis, asthma exacerbation, acute appendicitis). Accordingly, many children with severe illnesses will need transfers to other facilities. EDs without the inpatient resources necessary to care for children or children requiring admission need processes to efficiently stabilize and safety transfer children to facilities with pediatric resources.

**Table 3:** Results of national studies of pediatric emergency care

	**Athey ( *Pediatr Emerg Care *, 2001)**	**Gausche-Hill ( *Pediatrics *, 2007)**	**Current study**
Year of survey	1998	2003	2009
Survey methods	Mailed survey (100% response rate, n = 101)	Mailed survey (29% response rate, n = 1,489)	Telephone survey (85% response rate, n = 279)
Department of Pediatrics	33%	57%	36%
Pediatric Intensive Care Unit	10%	18%	11%
Separate pediatric ED	7%	6%	10%
Pediatric consultant	64%	NA	66%
Pediatric ED coordinator	NA	18% Physician; 12% nurse coordinator	13% Physician; 14% nurse coordinator

Abbreviations: *ED*, emergency department; *NA*, not available.

Clearly, much work is left to be done to promote and institute pediatric preparedness in US EDs. Our survey suggests that there has been no progress in the assignment of pediatric coordinators in EDs. The 2009 guidelines for preparedness to treat children in the ED identified the designation of a coordinator for pediatric emergency care as an important *first* step in ensuring readiness for children [7]; therefore, the lack of progress on this front suggests that there are many EDs that probably have not taken even the first steps towards preparedness. Previous studies have cited lack of awareness of the guidelines, in general, as a major reason for poor compliance [5,17]. Other possible barriers include organizational constraints or a lack of the necessary funding/resources.

Our survey focused on the ability of facilities to manage three common pediatric presentations to the ED. It did not specifically address preparedness for emergency responses to public health emergencies, such as disease epidemics or mass casualties, another important component of preparedness. A recent study found that 88% of EDs have memoranda of understanding in place to handle adult cases during public health emergencies, while 56% have memoranda of understanding in place to transfer children from their ED when inpatient beds are unavailable [18]. Ensuring that plans are in place to efficiently transfer children to facilities with inpatient resources during *non*-public health emergencies may facilitate planning for unexpected public health emergencies.

Our finding that an ED pediatric visit volume of ≥1 patient per hour and urban location were significant positive predictors of an emergency care pediatric coordinator is consistent with another study that examined factors associated with hospital preparedness for treating pediatric emergencies [19]. Burt and Middleton examined three domains of preparedness: pediatric services, expertise, and supplies. Their study found that, among other factors, hospitals that treat larger numbers of children in their EDs and that are located in metro areas are associated with better preparedness.

Based on the reported number of annual visits per year by children, EDs in our sample had a median daily visit volume of 11 pediatric patients (i.e., age less than 18 years old). This corresponds with previous findings that half of EDs provide care for fewer than ten children per day [5]. This relatively low daily volume may reduce the perceived need for guideline implementation. It also may make the presence of a pediatric inpatient service and a pediatric intensive care unit neither practical nor financially sustainable; however, all EDs must be prepared to receive, evaluate, and, at minimum, stabilize a child for transfer to a facility equipped to manage the illness or injury. In the absence of inpatient pediatric services, EDs can prepare for pediatric emergencies requiring hospital admission by developing processes to efficiently stabilize and safety transfer children to facilities.

Independent of annual ED pediatric visit volume, we found that EDs in a rural location were less likely than EDs in urban areas to have an emergency care pediatric coordinator. Previous work shows that staffing EDs in rural areas presents unique challenges [20] and that rural hospitals have less access to physicians trained in emergency medicine, pediatrics, or both [21]. Types of staffing may vary by time period (e.g., weekday vs. weekend), and rural hospitals may use a combination of medical staff and contracted coverage for their EDs, which may make the identification of an emergency care pediatric coordinator more difficult. In addition, there may be long distances between a rural ED and a hospital that admits critically ill pediatric patients, which makes it necessary for rural hospitals to stabilize and transfer these patients [21]. Given the unique challenges faced by rural EDs, rural EDs may require special efforts related to guideline communication and implementation.

There are renewed calls to improve the pediatric capabilities of EDs. In October 2010 the National Commission on Children and Disasters, an independent, bipartisan body established by the US Congress and President, recommended that states and hospital accrediting agencies ensure that all EDs adopt the 2009 guidelines [22]. Some efforts of this nature are underway. The Emergency Medical Services for Children program has facilitated the implementation of a recognition process for facilities with pediatric capabilities in several states [23-25]. These states have initiated the development of a categorization system to identify EDs that meet specific requirements deemed important for the management of pediatric emergencies. There is some evidence that EDs designated as pediatric-prepared have improved outcomes. An evaluation of Illinois facilities that received the designation of Emergency Department Approved for Pediatrics (EDAP) showed a significant reduction in mortality rates among patients 0–15 years old admitted from the ED for injury (13.7 deaths per 1,000 inpatients pre-EDAP vs. 11.0 deaths per 1,000 inpatients post-EDAP) [26]. Although these decreases may be due to multiple factors, we believe that awareness of pediatric emergency care needs from the facility designation process probably played a role.

Potential shortcomings of our study include that not all surveys were answered by nurse managers or ED directors, which could have influenced the accuracy of the data. However, our data largely correspond with previous studies of pediatric emergency care capabilities of EDs [3,5]. In addition, while our data suggest potential issues with EDs’ ability to successfully provide pediatric emergency services, we do not present data on whether this affects actual patient outcomes. An examination of outcomes was beyond the scope of this study, but is an important topic for future research. Finally, we did not assess the availability of age-appropriate equipment. We recognize that this is an important component in preparedness to treat children in EDs. Instead, we chose to focus on one of the three major aspects of pediatric emergency care capabilities (designated coordinator for the emergency care of children) because it allowed us to conduct the survey by telephone, an important component in achieving the high response rate (85%) that would yield the most accurate assessment of current pediatric care capabilities in US EDs.

## Conclusions

In summary, millions of children in need of emergent health care present to EDs at facilities that do not have specialized pediatric services or the ability to treat severe cases of common pediatric conditions. The lack of a significant increase in the availability of pediatric emergency services over time suggests a continued need for improvements to ensure access to emergency care for children.

## Abbreviations

CI: Confidence interval; ED: Emergency department; EDAP: Emergency department approved for pediatrics; IOM: Institute of medicine; NEDI: National emergency department inventory; OR: Operating room.

## Competing interests

The authors declare that they have no competing interests.

## Authors’ contributions

AFS helped design the study, provided study supervision, drafted the article, and interpreted the data. SAR drafted the article, and provided interpretation of the data. ALG acquired the data, provided critical revision of the manuscript, and provided administrative support. APS acquired the data, provided critical revision of the manuscript, and provided administrative support. JAE completed the data analysis and provided critical revision of the manuscript. CAC conceived of and helped design the study, provided critical revision of the manuscript, and provided overall study supervision. All authors gave final approval of the submitted manuscript.

## Supplementary Material

Additional file 1 Pediatric Emergency Medicine Survey. Click here for file
