# Supplementary material for: National survey of pediatric services available in US emergency departments
Source: Int J Emerg Med. 2013 Apr 24;6:13. doi: 10.1186/1865-1380-6-13 (PMC3639791; doi:10.1186/1865-1380-6-13)
Supplement: Additional file 1 — Pediatric Emergency Medicine Survey. [file 1865-1380-6-13-S1.pdf]

Pediatric Emergency Medicine Survey

1) In 2008, was your ED open:

a. 24 hours/day, 7 days/week? ☐1 Yes ☐0 No

b. 365 days per year? ☐1 Yes ☐0 No

If NO to either question, please explain: ____

2) Does your ED regularly manage common pediatric complaints <u>on site</u>, such as:

a. A 6-month old with bronchiolitis? (i.e., a lower respiratory tract infection of young children)
☐1 Yes, all severity levels; severe cases receive inpatient care at your hospital
☐2 Yes, mild to moderate cases only (outpatient care); severe cases are transferred to another hospital
(specify hospital name and city: ________________________________)
☐0 No

b. A 6-year old with an asthma exacerbation?
☐1 Yes, all severity levels; severe cases receive inpatient care at your hospital
☐2 Yes, mild to moderate cases only (outpatient care); severe cases are transferred to another hospital
(specify hospital name and city: ________________________________)
☐0 No

c. A 6-year old with acute appendicitis?
☐1 Yes, evaluate and transfer to the OR at your hospital (i.e., an in-house facility where surgical procedures are performed)
☐2 Yes, evaluate, but transfer to another hospital for surgery
(specify hospital name and city: ________________________________)
☐0 No

3) Although every ED would evaluate a critically ill patient of any age, some EDs predominantly treat adults, while others may predominantly treat younger patients (e.g., age <18 years). Does your ED have an age-related policy?

☐1 Yes (specify age range): ____________ ☐0 No, all ages seen

4) For EDs that regularly treat adults: **Does your ED have a separate "pediatric ED" (i.e., a dedicated ED area for children only)?**
☐1 Yes
☐0 No, children <12 treated alongside adults in the same general area
☐2 Not applicable (e.g., children's hospital)

If YES,
How many dedicated pediatric beds does your ED have? # PED ED BEDS ☐☐☐

5) What is the <u>total</u> number of beds in your ED # ED BEDS ☐☐☐
(exclude hallway and ED-based OBS unit, if applicable;
include dedicated pediatric beds, if applicable)

6) What is the total number of pre-designated hallway beds # HALLWAY BEDS ☐☐☐
(i.e., beds located in hallways used when standard rooms are at or near full occupancy.)

7) What is the total number of ED-based OBS unit beds # OBS BEDS ☐☐☐
(i.e., beds for ED patients who require monitoring for up to forty-eight hours.)

STAFFING

8) Do you have identified coordinators for pediatric emergency medicine in your ED? [check all that apply]
☐ Yes, physician coordinator(s)
☐ Yes, nurse coordinator(s)
☐ No

9) Is at least one <u>attending physician</u> (not resident) on duty <u>in the ED</u> 24 hours/day, 7 days/week? (exclude on-call physicians)

☐1 Yes ☐0 No

If NO,

When a physician is unavailable, is any physician available to the ED by two-way voice communication 24 hours/day, 7 days/week:

a. From within your hospital? ☐1 Yes ☐0 No

b. From outside of your hospital? ☐1 Yes ☐0 No

10) What percent of attending emergency physicians are board-certified or board-eligible by American Board of Emergency Medicine (ABEM), American Osteopathic Board of Emergency Medicine (AOBEM) or American Board of Pediatrics/Pediatric Emergency Medicine (ABP/Peds EM)? % BC/BE [| |]

11) Are the following consultants available in-person to the ED?

		Yes	No		On average, how long does the consultant take to arrive? 0-29 min	30-59 min	> 60 min	Is the consultant available 24 hours/day, 7 days/week? Yes	No
a.	Anesthesiologist	☐1	☐0	If YES →	☐0	☐1	☐2	☐1	☐0
b.	Cardiologist	☐1	☐0	If YES →	☐0	☐1	☐2	☐1	☐0
c.	General Surgeon	☐1	☐0	If YES →	☐0	☐1	☐2	☐1	☐0
d.	Neurologist	☐1	☐0	If YES →	☐0	☐1	☐2	☐1	☐0
e.	Neurosurgeon	☐1	☐0	If YES →	☐0	☐1	☐2	☐1	☐0
f.	Obstetrician-Gynecologist	☐1	☐0	If YES →	☐0	☐1	☐2	☐1	☐0
g.	Orthopedic Surgeon	☐1	☐0	If YES →	☐0	☐1	☐2	☐1	☐0
h.	Pediatrician	☐1	☐0	If YES →	☐0	☐1	☐2	☐1	☐0

12) Does your ED obtain consultation via video conferencing equipment? (e.g., video transmission to outside experts for evaluation of an acute stroke patient in your ED)

☐1 Yes (specify): ________________________________

☐0 No

NUMBER OF VISITS EACH YEAR

13) a. **What is the total number of patient visits at your ED each year?** [| | | | |]

NUMBER OF VISITS EACH YEAR BY CHILDREN AND ADULTS

b. **Does your ED use an age other than 18 years to distinguish between children and adults?**
If YES, specify cut-off: age < _____ years)

c. **How many visits by children (e.g., age <18) does your ED have each year?** [| | | | |]

d. **How many visits by adults (e.g., age 18+) does your ED have each year?** [| | | | |]

e. **What is the approximate date range used for these estimates?** **From** ____/____ (MM YY) **to** ____/____ (MM YY)

14) Is there a department of pediatrics in your hospital?

☐1 Yes ☐0 No

15) Does your hospital have a dedicated pediatric ICU? (i.e., a unique and physically separate facility from the ICU with its own staff that specializes in pediatric critical care)

☐1 Yes ☐0 No

If NO,
Where is the nearest dedicated pediatric ICU to which you would transfer critical care patients?

____ ☐ not sure

NAME OF HOSPITAL AND CITY

16) Approximate percent of all ED visits that led to admission, including OBS admissions (ED-based OBS unit + OBS status)

% ADMITTED [|] = % OBS [|] + % REGULAR [|] + % ICU [|]

THANK YOU!
